# A Genome-Wide Analysis of the LBD (LATERAL ORGAN BOUNDARIES Domain) Gene Family in *Malus domestica* with a Functional Characterization of *MdLBD11*

**DOI:** 10.1371/journal.pone.0057044

**Published:** 2013-02-28

**Authors:** Xiaofei Wang, Shizhong Zhang, Ling Su, Xin Liu, Yujin Hao

**Affiliations:** 1 National Key laboratory of Crop Biology, Shandong Agricultural University, Tai-An, Shandong, China; 2 National Research Center for Apple Engineering and Technology, Shandong Agricultural University, Tai-An, Shandong, China; 3 College of Horticulture Science and Engineering, Shandong Agricultural University, Tai-An, Shandong, China; Wuhan University, China

## Abstract

The plant-specific LBD (LATERAL ORGAN BOUNDARIES domain) genes belong to a major family of transcription factor that encode a zinc finger-like domain. It has been shown that LBD genes play crucial roles in the growth and development of *Arabidopsis* and other plant species. However, no detailed information concerning this family is available for apple. In the present study, we analyzed the apple (*Malus domestica*) genome and identified 58 *LBD* genes. This gene family was tested for its phylogenetic relationships with homologous genes in the *Arabidopsis* genome, as well as its location in the genome, structure and expression. We also transformed one *MdLBD* gene into *Arabidopsis* to evaluate its function. Like *Arabidopsis*, apple *LBD* genes also have a conserved CX_2_CX_6_CX_3_C zinc finger-like domain in the N terminus and can be divided into two classes. The expression profile indicated that apple *LBD* genes exhibited a variety of expression patterns, suggesting that they have diverse functions. At the same time, the expression analysis implied that members of this apple gene family were responsive to hormones and stress and that they may participate in hormone-mediated plant organogenesis, which was demonstrated with the overexpression of the apple *LBD* gene *MdLBD11*, resulting in an abnormal phenotype. This phenotype included upward curling leaves, delayed flowering, downward-pointing flowers, siliques and other abnormal traits. Based on these data, we concluded that the *MdLBD* genes may play an important role in apple growth and development as in *Arabidopsis* and other species.

## Introduction

LBD (LATERAL ORGAN BOUNDARIES domain) gene in *Arabidopsis* is a newly discovered and unique transcription factor family that has been assigned to this functional group on the basis of its nuclear localization and capacity to bind to a DNA motif [1], [2], [3], [4], [5]. Until now, *LBD* genes were found only in plant databases, indicating that this unique gene family may only regulate plant-specific processes [6]. The *LBD* gene family can be divided into two classes according to the structure of the LOB (LATERAL ORGAN BOUNDARIES) domain in the N terminus [6], [7]. Class I *LBD* genes contain a perfectly conserved CX_2_CX_6_CX_3_C zinc finger-like domain and an LX_6_LX_3_LX_6_L leucine zipper-like coiled-coil motif, while class II *LBD* genes only have a conserved zinc finger-like domain [6], [8]. The zinc-finger-like domain is presumably required for DNA binding, and the C-terminal leucine zipper-like sequence is probably involved in protein dimerization [6], [7] LBD proteins have varied expression patterns ranging from temporal to tissue differences, suggesting that they may function in diverse processes [6]. Numerous *LBD* genes are expressed at the adaxial base of plant lateral organs, and they play critical roles in lateral organ development during a plant’s growth [4], [6], [9], [10]. When observed under normal growth conditions, no obvious phenotype could be found in some loss-of-function *lbd* mutants, indicating that the *LBD* gene is functionally redundant or required during growth under specific environmental conditions [1], [6], [11].

By now, several members of the *LBD* family have been functionally identified in different species. Among class I *LBD* genes, the *LOB* gene encodes a DNA-binding protein and can interact with members of the bHLH (basic helix-loop-helix) family of transcription factors, while the interaction between bHLH proteins and *LOB* reduced the affinity of *LOB* for its consensus DNA motif [2]. *RA2* (Ramosa 2), a maize ortholog of *LOB*, has been shown to regulate reproductive growth [12], [13]. The *AS2*/*AtLBD6* gene influence the expression of class I KNOX genes through its interaction with MYB domain transcription factor AtAS1 and ERECTA, thereby regulating the establishment of leaf polarity [8], [14], [15], [16], [17], [18], [19], [20], [21], [22]. *AtASL1*/*AtLBD36* and *AtLBD12*/*AtASL5* have phenotypes that are similar to those found in *AtAS2*/*AtLBD6* overexpression plants [23].The maize *ZmLBD19* gene dimerize with the maize *AtAS1* ortholog *RS2* (*ROUGH SHEATH2*) and influence female gametophyte development [20], [24]. Together with *AtLBD16* and *AtLBD29*, *AtLBD18* is regulated by *AtARF7* and *AtARF19* and plays a role in lateral root initiation [1], [3], [5], [25], [26], [27], [28]. *Crl1*/*Arl1*, which is a homolog of *AtLBD29*, has a similar function in rice [29], [30], [31]. The *JAGGED LATERAL ORGANS*/*JLO* gene regulates *Arabidopsis* embryo and root development [32], [33]. The expression of *DDA1/LBD25* is reduced by exogenous auxin, and a *dda1* mutant change the number of lateral roots [34]. *AtLBD3*/*AtASL9* is induced by exogenous cytokinin treatment, indicating that this gene plays an important role in cytokinin-responsive biological events in plants [35]. *PtaLBD1* regulates secondary growth in poplar [36]. The *AtLBD20* gene takes part in jasmonate signaling in response to *Fusarium* wilt in *Arabidopsis* [37], *AtLBD1* may be involved in sustaining indeterminate cell fate of SAMs in Cockscomb [38]. For class II *AtLBD* genes, *AtLBD37*, *AtLBD38* and *AtLBD39* are induced by nitrate and involved in anthocyanin synthesis and nitrate metabolism [11]. *AtLBD41* is proposed to be involved in leaf dorsoventral determination [10]. Extensive studies of the *AtLBD*s in various plant species have provided a better understanding of this gene family. As of this publication, 43 *Arabidopsis* LBD proteins and 35 of rice homologs have been found [6], [39]. In maize and poplar, the *LBD* gene family consists of 43 and 57 members [4], [40], [41], respectively. Most recently, 3 *LBD* genes are considered as strong candidates of *Co* locus controlling columnar growth habit in apple [42], suggesting that *LBD* genes may play an important role in the control of apple tree architecture. However, it is yet unknown how many *LBD* genes in apple genome and how about their genomic organization in 17 apple chromosomes.

In this study, a genome-wide survey of the *LBD* gene family was conducted using the apple genome database [43]. The genome sequences of all apple *LBD* genes were identified with online software. Their genome structure, chromosome distribution and expression patterns were analyzed *in silic*o. Subsequently, real time PCRs and semi-quantitative RT-PCRs were carried out to confirm the expression patterns in different organs and in response to hormones and various stimuli. In addition, *MdLBD11* was functionally characterized in transgenic *Arabidopsis*. This study will serve as a foundation for future research into the functional roles of *MdLBD* genes.

## Results

### The Identification and Annotation Information of the *LBD* Genes in Apple

To identify the LBD proteins in apple, a local BLAST program and the Hidden Markov Model of the SMART and Pfam tools were used, and a total of 58 *LBD-like* genes from the entire apple genome were identified. We discovered that all of these LBD-like protein sequences possess a modular structure and conserved LOB motifs. As a result, 58 putative MdLBD proteins were found (Table 1). And we named each gene on the basis of its location on the chromosome. We noted the gene identifier, genomic position, *pI* (isoelectric point), length of amino acid, and protein size in Table 1. From the table, we can see that all the identified LBD genes encode proteins ranging from 130 (*MdLBD47*) to 431 (*MdLBD26*) amino acids along with a protein mass from 14.9 kD to 44.3 kD and protein pIs ranging from 4.63 (*MdLBD51*) to 9.69 (*MdLBD47*).

**Table 1:** Gene search and genetic nomenclature based on chromosomal position.

Gene identifier	Gene name	Genomic position	Size(aa)	Mass(Da)	pI
MDP0000151784	*MdLBD1*	chr01∶14333863.14334877	5.83	24997.89	230
MDP0000123013	*MdLBD2*	chr01∶14336168.14337182	5.83	24997.89	230
MDP0000879799	*MdLBD3*	chr01∶14339802.14340857	5.93	25224.84	232
MDP0000319331	*MdLBD4*	chr01∶16654621.16655986	5.31	22966.92	212
MDP0000123797	*MdLBD5*	chr01∶18212023.18212767	5.05	24303.39	216
MDP0000220708	*MdLBD6*	chr01∶21271862.21272392	8.37	19277.78	176
MDP0000194766	*MdLBD7*	chr01∶21348445.21349196	6.36	18781.5	168
MDP0000278334	*MdLBD8*	chr01∶21731069.21732173	9.06	28901.21	261
MDP0000167580	*MdLBD9*	chr01∶21747577.21749606	9.21	21690.61	189
MDP0000193830	*MdLBD10*	chr02∶15182073.15182800	8.26	19754.84	181
MDP0000562305	*MdLBD11*	chr02∶15438443.15439345	8.22	32845.8	300
MDP0000305695	*MdLBD12*	chr02∶17692925.17693725	9.56	27020.33	245
MDP0000317227	*MdLBD13*	chr02∶17696966.17697805	9.19	30751.13	279
MDP0000254224	*MdLBD14*	chr02∶22462044.22466145	7.22	28591.6	273
MDP0000429067	*MdLBD15*	chr02∶29675335.29678584	8.18	24836.09	228
MDP0000861160	*MdLBD16*	chr03∶5441634.5442565	6.42	19506.22	174
MDP0000318244	*MdLBD17*	chr03∶9623522.9625101	5.51	23040.14	213
MDP0000218986	*MdLBD18*	chr03∶9636639.9638218	5.51	23040.14	213
MDP0000943252	*MdLBD19*	chr04∶2582353.2582886	8.03	19402.92	177
MDP0000155138	*MdLBD20*	chr04∶2667092.2667817	5.8	18924.55	169
MDP0000643326	*MdLBD21*	chr04∶22958911.22959657	7.25	27154.82	248
MDP0000133546	*MdLBD22*	chr04∶22964675.22965421	6.94	25710.46	235
MDP0000294210	*MdLBD23*	chr05∶4850682.4853618	6.08	43454.21	394
MDP0000197526	*MdLBD24*	chr05∶7149837.7150580	6.29	27026.23	247
MDP0000777060	*MdLBD25*	chr05∶7775605.7776396	6.04	28568.98	263
MDP0000284409	*MdLBD26*	chr05∶11232036.11242489	8.54	47730.03	431
MDP0000261146	*MdLBD27*	chr05∶25844239.25845114	8.64	25111.65	224
MDP0000247079	*MdLBD28*	chr05∶25883261.25884136	8.81	25148.14	224
MDP0000753623	*MdLBD29*	chr06∶11066243.11066769	8.28	15775.72	139
MDP0000130659	*MdLBD30*	chr06∶19262057.19262656	8.55	21504.55	199
MDP0000153290	*MdLBD31*	chr06∶20514370.20515613	6.14	41855.15	369
MDP0000697271	*MdLBD32*	chr07∶5286028.5286741	8.38	26658.38	237
MDP0000200379	*MdLBD33*	chr07∶5319673.5320383	8.58	26490.15	236
MDP0000223109	*MdLBD34*	chr07∶17590374.17591071	5.88	20009.99	172
MDP0000822986	*MdLBD35*	chr07∶20982923.20984041	6.19	25177.01	231
MDP0000276955	*MdLBD36*	chr08∶8082319.8082765	8.26	16593.61	148
MDP0000487015	*MdLBD37*	chr08∶12183285.12186216	8.42	31397.98	288
MDP0000747679	*MdLBD38*	chr08∶12200557.12203488	8.42	31397.98	288
MDP0000190306	*MdLBD39*	chr09∶2721683.2722786	5.92	34682.85	257
MDP0000136037	*MdLBD40*	chr09∶5735343.5736376	8.45	32638.87	305
MDP0000121821	*MdLBD41*	chr09∶12599870.12600742	6.36	17816.15	158
MDP0000139025	*MdLBD42*	chr10∶8653279.8654145	7.53	23785.97	315
MDP0000268890	*MdLBD43*	chr10∶21049069.21053744	7.64	19443.01	176
MDP0000121203	*MdLBD44*	chr10∶24617746.24620393	9.06	25207.91	230
MDP0000430686	*MdLBD45*	chr10∶27855557.27861011	6.55	44319.96	395
MDP0000265429	*MdLBD46*	chr11∶5634618.5635555	6.78	19659.53	176
MDP0000277239	*MdLBD47*	chr12∶24251934.24252987	9.69	14936.27	130
MDP0000273432	*MdLBD48*	chr12∶31634852.31637028	9.04	33470.66	300
MDP0000254288	*MdLBD49*	chr14∶24069525.24070194	5.08	23696.41	311
MDP0000794101	*MdLBD50*	chr15∶1713236.1714021	7.46	24529.95	226
MDP0000133936	*MdLBD51*	chr15∶4034464.4035214	4.63	24368.26	218
MDP0000239820	*MdLBD52*	chr15∶25823210.25825670	8.72	29926.14	275
MDP0000151330	*MdLBD53*	chr15∶25823768.25825218	9.03	36853.69	335
MDP0000257227	*MdLBD54*	chr15∶33651689.33653060	6.07	35261.09	314
MDP0000848210	*MdLBD55*	chr17∶2027358.2027888	6.89	19516.68	176
MDP0000131964	*MdLBD56*	UN	8.38	23121.3	213
MDP0000145761	*MdLBD57*	UN	8.38	22915.01	211
MDP0000216231	*MdLBD58*	UN	5.68	42576.48	375

### Phylogenetic and Gene Structure Analysis of the *LBD* Genes

**Figure 1:**
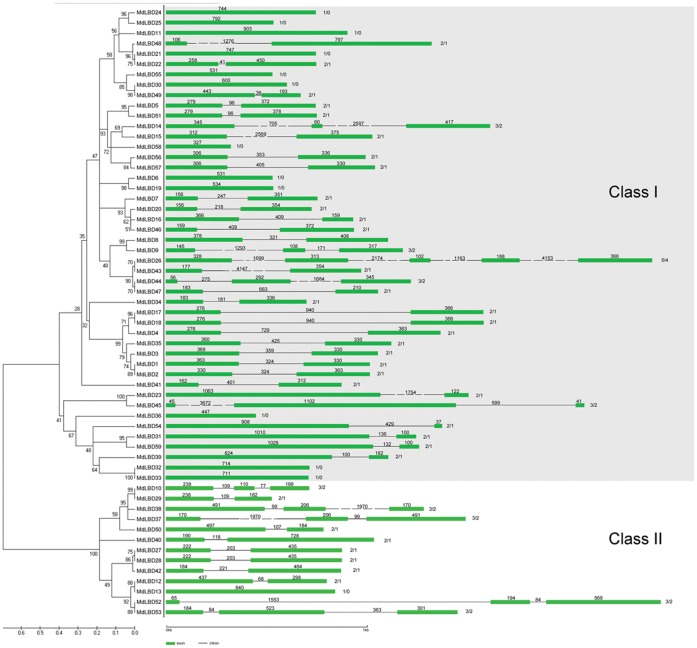
The phylogenetic tree and gene structure analysis of the MdLBD proteins. The amino acid sequences of the LBD proteins were aligned with ClustalX, and the phylogenetic tree was constructed using the neighbor-joining method of MEGA 5.0 software. Each node is represented by a number that indicates the bootstrap value for 1000 replicates. The scale bar represents 0.1 substitutions per sequence position (left). The right side illustrates the exon-intron organization of the corresponding LBD genes. The exons and introns are represented by the green boxes and black lines, respectively. The numbers indicate the length of the exons or introns. The scale bar represents 1000 bp (right).

To evaluate the evolutionary relationships among the 58 apple LBD proteins, we performed a phylogenetic analysis based on their full length amino acid sequences. We identified two subfamilies (class I and class II) as being monophyletic (Fig. 1), and 11 sister pairs of paralogous *LBD*s (*MdLBD5/51, MdLBD6/19, MdLBD8/9, MdLBD24/25, MdLBD30/49, MdLBD17/18, MdLBD23/45, MdLBD31/59, MdLBD31/59, MdLBD32/33* and *MdLBD10/29*) were found, 10 of which had very strong bootstrap support (>90%). Our results suggest a clear paralogous pattern of *LBD* gene divergence by gene duplication for the apple.

Structural analyses were intended to provide valuable information concerning duplication events when interpreting phylogenetic relationships within gene families. Thus, we analyzed the exon/intron structures of LBD family genes (right panel in Fig. 1). In apples, the exon number ranged from 1 within twelve genes to 5 in *MdLBD26*. Most *MdLBD* genes shared 1, 2 or 3 exons. Specifically, 35 genes had two exons, 12 genes had one exon, and only one gene had 5 exons. Most members within the same subgroup shared a similar intron/exon structure and gene length. The conserved intron/exon structure in each subgroup supported their close evolutionary relationship and the stated classification of subfamilies.

**Figure 2:**
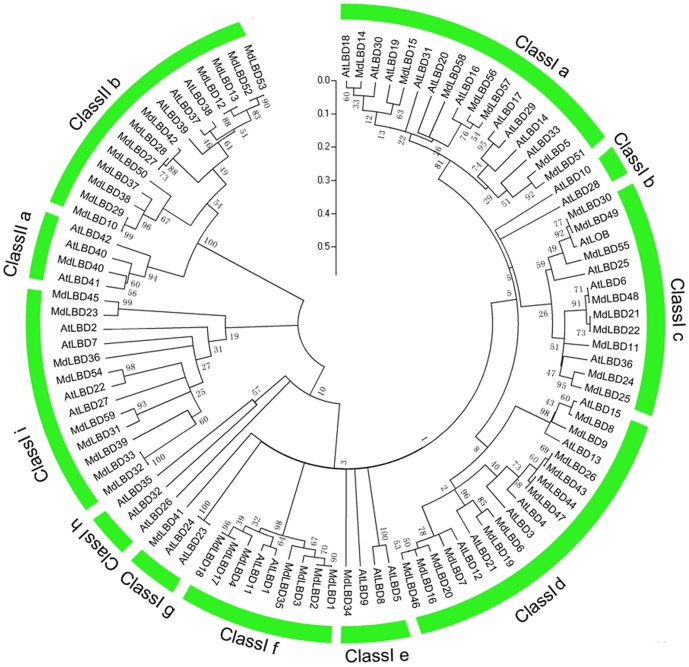
The phylogenetic analysis of *LBD* genes in apple and *Arabidopsis*. The amino acid sequences of the LBD proteins were aligned with Clustal X, and the phylogenetic tree was constructed using the neighbor-joining method of MEGA 5.0 software. Each node is represented by a number that indicates the bootstrap value for 1000 replicates. The scale bar represents 0.1 substitutions per sequence position. Each LBD subfamily is indicated by an arc.

To examine the evolutionary patterns of *MdLBD* with those of *Arabidopsis* and then group them into established subfamilies, a phylogenetic tree was generated with full length protein sequences (Fig. 2). As in rice and *Arabidopsis*, the *MdLBD* clearly fell into two classes, class I and class II, which had 45 and 13 separate genes relative to 37 and 6 genes in *Arabidopsis*, as shown in Fig. 1. The presence of twice as many class II apple LBD genes compared with *Arabidopsis* indicates that this class of gene may have more functions in apple development. Class I and class II families were further divided into 8 and 2 groups. In class I, the subgroups were named from class Ia to class Ii, while class IIa and class IIb comprised the class II group. Three *Arabidopsis* LBD genes (*AtLBD40,AtLBD41*,*AtLBD42*) that were clustered into class IIa corresponded with only one LBD gene (*MdLBD40*) in apple. On the other hand, there were 12 apple LBD genes grouped in class IIb with only three genes (*AtLBD37*, *AtLBD38*, and *AtLBD39*) in *Arabidopsis*. It is reported that *AtLBD37*, *AtLBD37*, *AtLBD38* and *AtLBD39* function as transcript regulators in response to nitrate and regulate gene expression relative to anthocyanin biosynthesis along with nitrate uptake and transport [11]. We conclude that class IIb LBD genes may play a broader function in apple.

### Chromosomal Location Analysis of *MdLBDs*

**Figure 3:**
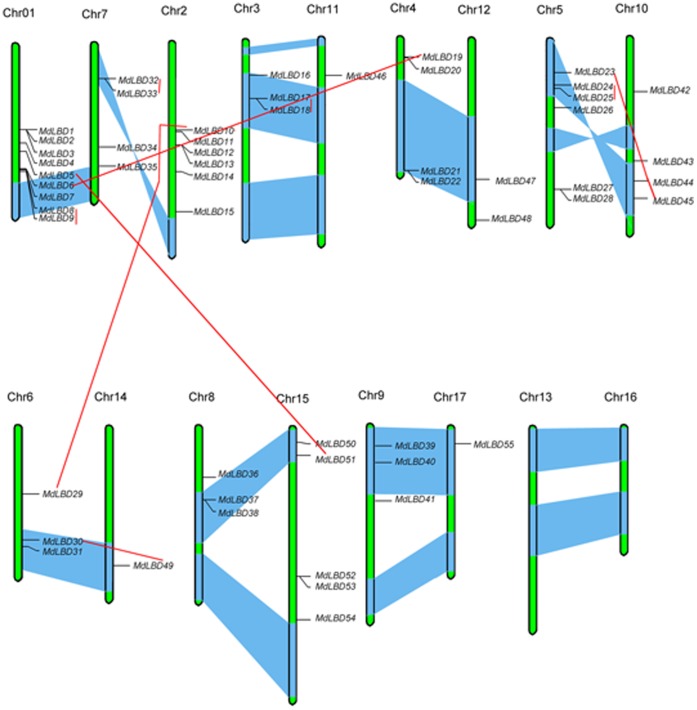
The chromosomal mapping analysis of the *LBD* gene family in apple. The scale bar represents a 10.0 Mb chromosomal distance. The chromosome number (Chr01-Chr17) is indicated at the top of each chromosome. To simplify the presentation, we named the putative LBD genes from MdLBD1 to MdLBD58 on the basis of the LBD family and gene order on the chromosomes from Chr01 to Chr17, respectively. Segmented duplicate homologous blocks are indicated with a blue shadow. Sister paralogous pairs are indicated by a red line.

Fifty-five genes were located in 15 out of 17 chromosomes, and 3 *MdLBD* genes (*MdLBD56, MdLBD57 and MdLBD58*) were localized to unassembled genomic sequence scaffolds and thus were not mapped to any particular chromosome (Fig. 3). Chromosome 01 encompassed the largest number with 9 *MdLBD* genes, followed by Chr02 and Chr05 (6 genes per chromosome). Five *MdLBD* genes were distributed in Chr15, four genes were distributed in Chr04, 7 genes were distributed in Chr03, 10 genes were distributed in Chr06, and three genes were distributed in Chr08. Two *LBD* genes were identified in Chr12, while only one gene was located on Chr11, 14 and 17. In addition, further investigation showed that the distribution of each type of *LBD* genes was significantly irregular (Fig. 3), while no *MdLBD* genes were located on Chr13 or Chr16. Among them, a total of 21 genes were found in the segmental duplication blocks (Fig. 3), and five homologous pairs (*MdLBD17/18*, *MdLBD24/25, MdLBD23/45, MdLBD32/33* and *MdLBD30/49*) were definitely located on the duplicated blocks.

### Sequence Alignment and Conserved Motifs of *MdLBD* Genes

**Figure 4:**
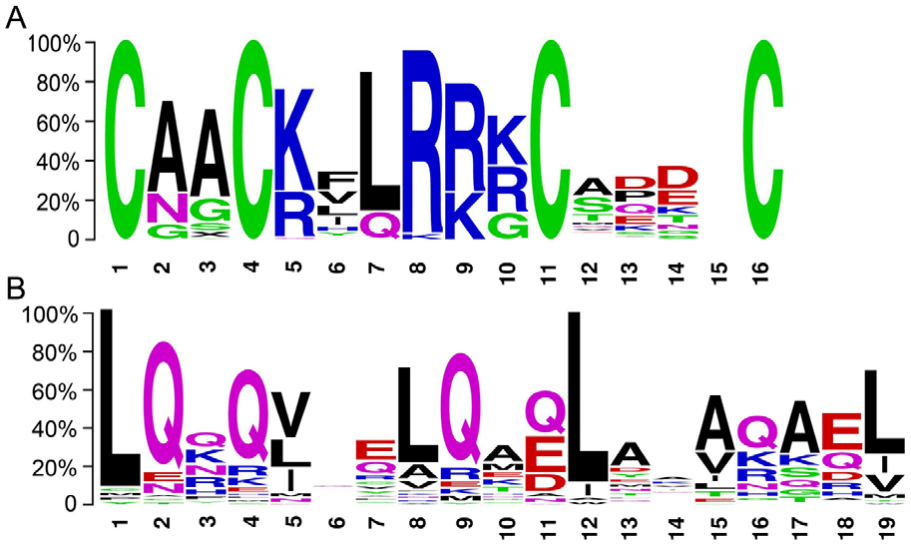
Conserved domains of MdLBDs gene family. A. The CX_2_CX_6_CX_3_C zinc finger-like domain sequence logos. B. The LX6LX3LX6L leucine zipper-like domain sequences Logos. Sequence alignment of two domains by ClustalX and conserved motifs Logos was performed by the WebLogo program.

In *Arabidopsis*, the *LBD* genes had a conserved LOB domain in the N terminus of the genes, and there were two conserved blocks in the LOB domain of the class I proteins, that is, the C block and GAS block. To identify conserved domains within the *MdLBD* genes, we performed an alignment within all of the *MdLBD* genes and a separate one for the class I and class II protein sequences. As with the *AtLBD* genes, multiple sequence alignment showed that all 58 predicted MdLBD protein sequences had a completely conserved CX_2_CX_6_CX_3_C zinc finger-like domain while a LX6LX3LX6L leucine zipper-like domain existed only in 17 of the class I *LBD* genes (Fig. 4; Fig. S1). In contrast with the *AtLBD* genes, the *MdLBD* genes had completely conserved H/G amino acids at the GAS block. The GAS block ended with a DPVYG motif but not with a DP (V/I) YG motif.

### Spatial and Temporal Expression and Gene Response Analysis of some of the *MdLBD* Genes

**Figure 5:**
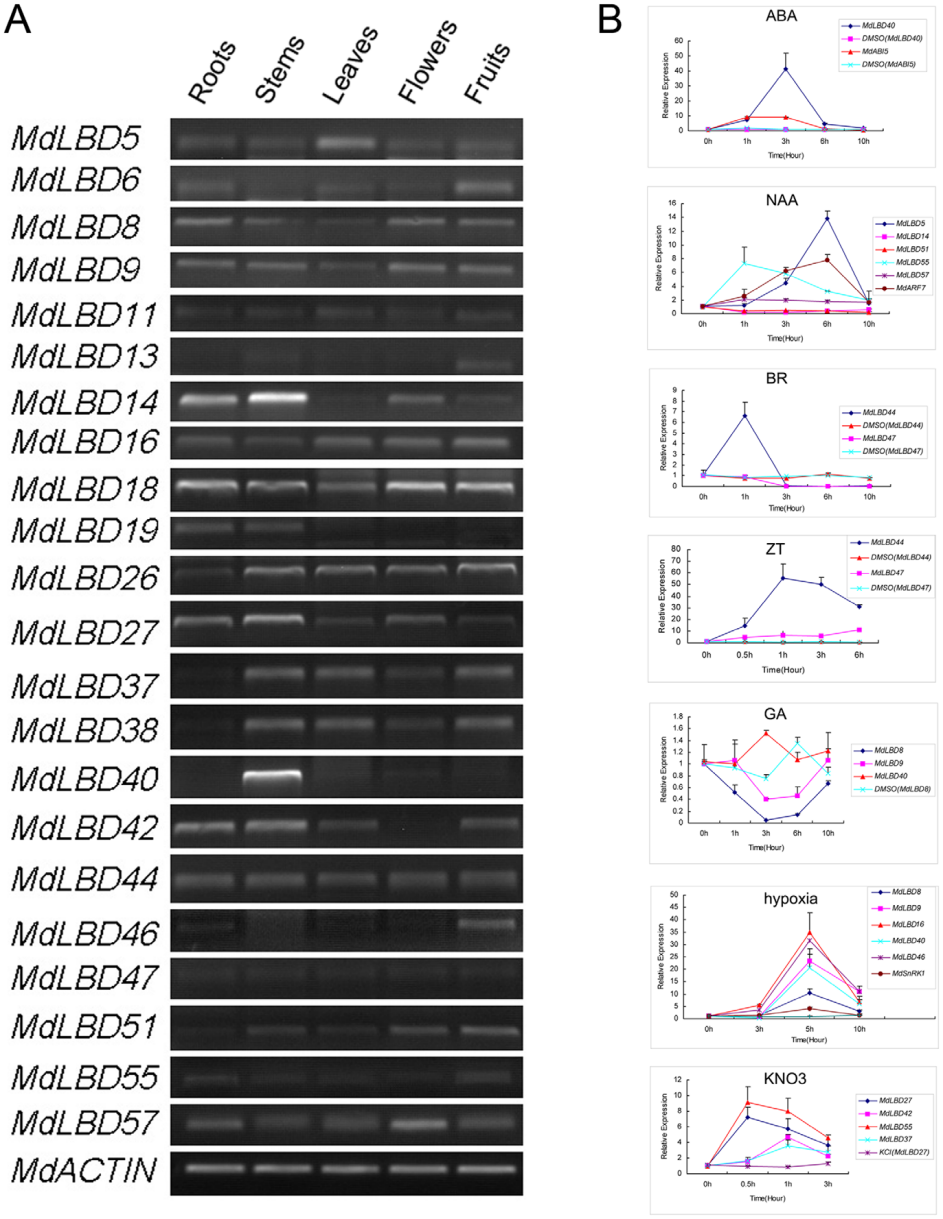
Tissue-specific expression profiles and gene response for the *MdLBD* genes. A. Tissue-specific expression profiles of *MdLBD* genes. Expression levels of *MdLBD* genes were examined by semi-qRT-PCR in apple roots (R), stems (S), leaves (L), flowers (FL) and fruits (F). The *MdACTIN* was performed as an internal control. B. QRT-PCR analysis of *MdLBD* genes in response to multiple treatments. *MdLBD* genes expression treated by 15 mM KNO_3_ (15 mM KCl was treated as a control), hypoxia, and 100 µM of ABA, NAA, 6-BA, GA and BR, the *MdACTIN* was performed as an internal control.

Semi-quantitative RT-PCR analysis was used to investigate the expression patterns of some of the *MdLBD* genes in the following organs: roots, stems, leaves, flowers and fruits. We found that transcripts of *MdLBD* genes could be detected in all tissues (Fig. 5A). Transcripts of *MdLBD19* were detected mainly in roots and leaves, and transcripts of *MdLBD40* were predominantly present in stems. Transcripts of *MdLBD14* could be detected in all tissues except the leaves, and the expression of *MdLBD42* could be detected in all organs except in flowers (Fig. 5A).

It was discovered that *AtLBD* genes can respond to different treatments, such as auxin, cytokinin, and nitrate. Based on the existing microarray experiments that were compiled in Genevestigator, we found that they also responded to the hormones ABA (abscisic acid), GA (gibberellin) and BR (brassinosteroid); the abiotic stress of hypoxia; and biotic stress from Pst (*Pseudomonas syringae*) (Fig. S2). We then analyzed the gene expression of *MdLBD* gene homologs corresponding to *AtLBD* genes. Bioinformatics analysis was used to predict the homologous gene in apple in comparison to *Arabidopsis*, although some differences existed between the two plants. RT-PCR analysis was performed to determine mRNA expression levels. We selected 21 genes that showed high homology with the *Arabidopsis* gene for expression assays under hormone treatment with NAA (1-naphthlcetic acid), GA (gibberellins), 6-BA (6-Benzylaminopurine), ABA (abscisic acid), and BR (brassinolide). In the RT-PCR assay, almost all detected *MdLBD* genes were especially responsive to one or more treatments (Fig. 5B). For example, the relative transcript level of *MdLBD40* was induced by approximately 40-fold after 3 hours of treatment with ABA when compared with the control (which was treated with DMSO), and a 9-fold increase was induced in *MdABI5*, a marker gene responding to ABA, which suggests that *MdLBD40* was ABA-responsive. Transcripts also increased by 14-fold, 7-fold and 2-fold for *MdLBD5*, *MdLBD55* and *MdLBD57*, respectively, at their peak levels when NAA was applied. Slightly down-regulated with *MdLBD14* and *MdLBD51*, *MdARF7* here was used as a marker gene. The BR induced the *MdLBD44* gene by more than 6-fold but repressed *MdLBD47* rapidly. *MdLBD44* and *MdLBD47* can also be induced by ZT by no less than 50-fold and 10-fold, respectively, at the peak. In response to GA, the expression of *MdLBD8* had a rapid reduction, while no significant change took place in the transcription of *MdLBD9* and *MdLBD40*. There were 10-fold to 35-fold changes for *MdLBD8*, *MdLBD40*, *MdLBD9*, *MdLBD46* and *MdLBD16* after about 5 h of exposure to hypoxia. *MdSnRK1* was used as a control. Nitrate-induced expression showed a maximum of 3-fold to 9-fold increase in *MdLBD55*, *MdLBD42*, *MdLBD27* and *MdLBD37* genes at the peak. These results indicate that the function of *LBD* genes may be conserved between different species. Various kinds of regulating hormone treatments suggest a potential function for the *MdLBD* gene in hormone-mediated plant organogenesis.

### *MdLBD11* has a Similar Function to the Homologous *Arabidopsis* Gene

**Figure 6:**
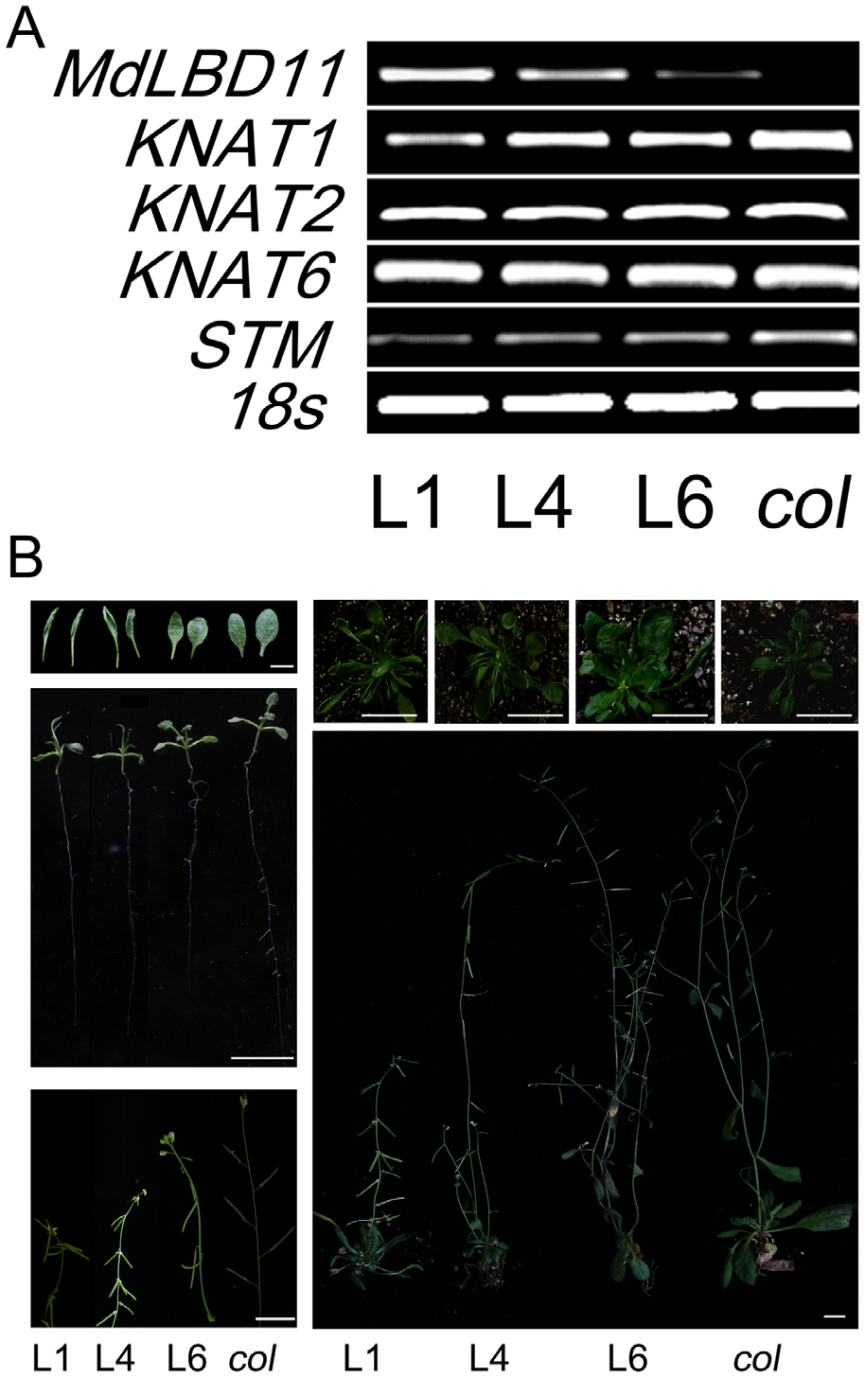
Ectopic expression of MdLBD11 changes the phenotype in transgenic *Arabidopsis.* A. Expression analysis of *MdLBD11* and *KNOX* genes in transgenic *Arabidopsis.* Expression levels of *MdLBD11* and *KNOX* genes were detected in transgenic *Arabidopsis* in comparison with wide type lines, and three transgenic lines were selected for expression analysis. B. Morphological characteristics of transgenic *Arabidopsis.* Two week-old rosettes (upper), one month-old (middle) and two month-old (bottom) transgenic *Arabidopsis* were compared with wide type lines. Bars = 1 cm.

In order to know more about the biological functions of *MdLBD* genes, *MdLBD11* was chosen for functional characterization. According to the sequence alignment and phylogenetic analysis, *MdLBD11* was highly similar to *Arabidopsis AtASL1* and *AtAS2* (Fig. S3). First, its cDNA was introduced into *Arabidopsis*. Consequently, three *Arabidopsis* transgenic lines were obtained with different ectopic expression levels (Fig. 6A). When compared with the WT control, all transgenic lines exhibited abnormal phenotypes in relation to vegetative development and growth, including reduced plant stature, short petioles and small and upward curled leaves. Meanwhile, transgenic plants also experienced long juvenility, and their downward-pointing flowers and siliques had short pedicels (Fig. 6B). The extent of abnormal phenotypes was positively correlated with the expression level of *MdLBD11* in transgenic lines, indicating that the ectopic expression of *MdLBD11* resulted in the transgenic phenotypes.

**Figure 7:**
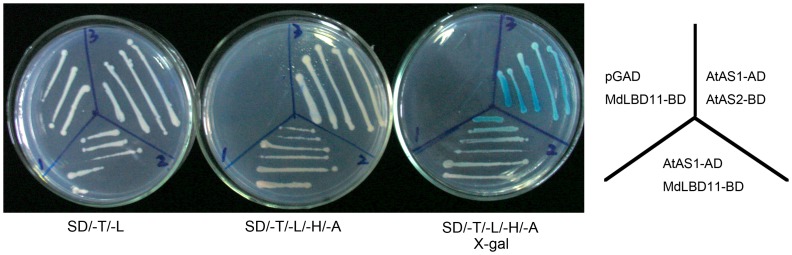
MdLBD11 interacts with AtAS1 in a yeast two-hybrid assay. Yeast strains containing pGAD-AtAS1, pGBD-MdLBD11, and pGBD-AtAS2 were assayed for LacZ expression, pGAD-AtAS1 in combination with pGBD-AtAS2 was used as a positive control, and pGAD with pGBD-MdLBD11 was used as a negative control. Yeast grew on SD/−T/−L medium to select for both the bait and prey proteins (left). SD/−T/−L/−A/−H media allow the growth of only positively interacting clones. SD/−T/−L/−A/−H media plus X-gal allow the growth of only the positively interacting clones.

Furthermore, to examine how *MdLBD11* regulates plant development and growth, yeast two-hybrid assay was conducted. The result showed that MdLBD11 interacted with *Arabidopsis* AtAS1 (Fig. 7). Meanwhile, the expressions of the class I KNOX genes were analyzed in *MdLBD11* overexpression lines. The results showed that all transgenic lines produced less *KNAT1* and *STM* transcripts than the WT control, with no obvious transcription difference in *KNAT2* and *KNAT6* (Fig. 6A), suggesting that *MdLBD11* regulated plant development and growth, at least partially if not completely by modulating class I KNOX gene expression, just as *AtAS2* and *AtASL1* in *Arabidopsis*. Furthermore, the results indicated that *MdLBD11* functioned with a conserved mechanism like its *Arabidopsis* counterparts *AtAS2* and *AtASL1*.

## Discussion

### Characterization of the Apple *LBD* Gene Family

*LBD* genes belong to a newly found unique transcript factor family. They ubiquitously exist in higher plants and may regulate plant-specific processes [4], [6]. Based on a global survey through the released apple genome database, 58 *MdLBDs* were identified from 57,386 annotated genes in apple genome [43]. Each of them was featured with a conserved LOB domain which ubiquitously exists in *LBD* genes of other plant species [4], [6]. They were localized to 15 chromosomes, except chr13 and chr16 out of the total of 17 apple chromosomes. Meanwhile, the cDNA of *MdLBD11L* was isolated with a RACE approach. Its nucleotide sequence is highly similar to that of *MdLBD11* cDNA. However, there is no genomic sequence information found corresponding to *MdLBD11L* in apple genome database. Similarly, 2 out of 3 *MdLBD* candidates for *Co* locus can not be found in apple genome database [42], suggesting that there may be more other *MdLBDs* existing in the unknown genomic gaps. In fact, only 81.3% genomic sequences are overlapped on the apple genome [43].

The *LBD* gene families contain 43, 35 and 35 members in *Arabidopsis*, rice, and maize, respectively [6], [39], [40]. However, apple genome has at least 58 *MdLBD*s. Therefore, *LBD* gene family evolutionarily expanded in apple, maybe due to the chromosome duplication. Many angiosperms undergo whole genome duplication events. The recent gene duplication events were the most important for the rapid expansion and evolution of gene families [44]. In apple, the chromosome homologies derived from the recent genome-wide duplication allow inference of the cytological events that have led to the number and composition of the extant apple chromosomes, from a putative nine-chromosome ancestor to the 17-chromosome [43]. In this study, we found 11 sister pairs of paralogous *LBDs*. Among them, 5 homologous pairs were definitely located on the duplicated blocks (Fig. 3). These data support that the chromosome duplication events occur in apple genome.

### Phylogenetic Analysis and Evolution of Apple LBD Genes

Phylogenetic analysis and evolutionary relationship of the LBD proteins have been intensively studied in *Arabidopsis* and rice [6], [39], which divides the *LBD* gene family into Class I and class II [4], [6], [7]. Here phylogenetic trees combining apple and *Arabidopsis* LBD proteins were constructed to analyze the relationship of 58 apple LBDs with their *Arabidopsis* counterparts. As a result, a total of 101 apple and *Arabidopsis* LBDs were divided into 10 clades, among of which 2 clades, i.e. class Ib and class Ih, exist only in *Arabidopsis* (Fig. 2). Therefore, 58 *MdLBDs* were distributed in the other 8 clades. In *Arabidopsis*, *LBD* genes in Class IIb are involved in the regulation of nitrate uptaking and anthocyanin synthesis [11]. In this study, it was found that Class IIb has 12 apple and 3 *Arabidopsis* LBD members, respectively (Fig. 1; Fig. 2). Apple has 4-fold more LBD members in class IIb than *Arabidopsis*, suggesting that woody apple tree evolutionarily needs more class IIb *LBD* genes to adapt barren soil and to regulate complex processes of fruit coloration. This may be an evolution diversity of *LBD* genes between herbaceous *Arabidopsis* and woody apple plants, just like between herbaceous rice and maize and woody poplar [39], [40], [41].

Structural analysis is a useful tool to mine valuable information concerning duplication events and phylogenetic relationships within gene families. In apples, the exon numbers of *LBD* genes ranged from 1 to 5, with more than half of them containing 2 exons. This is similar in *Arabidopsis* and rice, suggesting that the genetic evolution of the *LBD* genes structure is conserved in different angiosperms. Besides, most of the *MdLBD* members within the same clade share a similar intron/exon structure and gene length (Fig. 1), indicating their close evolutionary relationship.

So far, all known LBD proteins that are detected in various species have a conserved zinc finger-like domain, while a leucine zipper-like coiled-coil motif is found only in the majority of class I LBD proteins [4.6]. It has been predicted that Zinc finger-like motifs in the LOB domain may function as a DNA-binding domain, and the C-terminal leucine zipper-like motif may function in a probable protein-protein interaction [4]. Moreover, the incomplete leucine zipper-like coiled-coil motif is part of the class II LBD proteins. It is believed that this domain is misfunctional for protein-protein interaction. Actually, members of MYB and bHLH proteins have been found to interact with *Arabidopsis LOB*, a class I LBD protein [2]. We have also demonstrated that *MdLBD11* and *MdLBD11L*, which are two class I *LBD* genes, interacted with MYB transcription factor AtAS1 (Fig. 7; Fig. S4). A yeast two-hybrid assay was used to screen the potential interaction proteins with several class II *MdLBD* genes (*MdLBD12*, *MdLBD27*, *MdLBD52*), and as predicted, no proteins could be identified.

### Expression Analysis Indicated *LBD* Genes may Play Important Roles during Plant Growth and Development

Phytohormones have long been found to play a critical role in normal plant growth and development. Auxin, cytokinin and GA are the major developmental growth regulators, whereas ABA, ETH and JA are often implicated in stress responses. Members of LBD genes in *Arabidopsis*, rice and other species has been found respond to different treatments, such as auxin, cytokinin, GA and nitrate and they playe a key role in plant organ boundary definition by influencing the content or transport of the nutrient and hormone [1], [4], [11], [17], [35], [45]. In *Arabidopsis*, *AtLBD3* represss the cytokinin content and was directly activated by type-B ARR, indicating that it is a primary target of the cytokinin signal transduction pathway [35]. *AtLBD16* and *AtLBD28* link the auxin signal transduction cascade and are directly regulated by *AtARF7* and *AtARF19*, which are two major auxin response genes, through the auxin responsive elements (AuxRE) and then regulate embryonic and vascular patterning [1], [3], [25], [45]. *AtLBD40* is reported to be downregulated by gibberellin and upregulated by DELLA proteins [46]. In addition to transcript regulation, class I LBD proteins can also interact with some MYB and bHLH proteins to regulate plant organogenesis [2], [47]. These results confirm that *LBD* genes can regulate plant organ differentiation at the transcript and protein levels. *LBD* genes were also nutrient- and stress-responsive, revealing that they may regulate nitrogen metabolism and abiotic and biotic responses.

It was also found that the expressions of *LBD* genes responded to ABA and BR, hypoxia stress and Pst (*Pseudomonas syringae*) infection in model plants based on the online microarray database (Fig. S2), as confirmed by RT-PCR analysis in apple (Fig. 5B). Therefore, *LBD* genes responded to various phytohormones in apple. Furthermore, 3 apple *LBD* genes may be involved in the regulation of columnar growth habit [42]. As is well known, several *LBD* proteins interact with MYB and bHLH transcription factors which regulate organogenesis in higher plants [2], [14], [16], [17]. Therefore, it is reasonable to suppose that *LBD* genes may be involved in the regulation of plant organ boundary definition and shoot branching associated with various hormones by interacting with specific MYB or bHLH transcription factors. In addition, some loss-of-function *lbd* mutants do not exhibit abnormal development and growth [1], [6], [11], suggesting a functional redundancy of those *LBD* genes. Meanwhile, *LBD* genes clustered into the same clade show different expression patterns (Fig. 5; Fig. S5), suggesting a functional specificity for each *LBD* gene even in the same clade [11].

### *LBD* Gene may have the Conserved Function to the Homologous *Arabidopsis* Gene

*MdLBD11* transgenic *Arabidopsis* exhibited abnormalities both in vegetative and reproductive growth, which is highly similar to those observed in *AtASL1* and *AtAS2* overexpression transgenic *Arabidopsis* lines. This indicates that *MdLBD11* has functions similar to *AtASL1* and *AtAS2*. It is known that AtAS2 interacts with MYB transcription factor AtAS1 to regulate the expression of downstream class I KNOX genes, underlying its biological functions in *Arabidopsis* [8], [14], [16]. Interestingly, MdLBD11 also interacted with *Arabidopsis* AtAS1 and regulated the expression of KNOX genes (Fig. 6A; Fig. 7), indicating that *MdLBD11* functions in a way similar to its *Arabidopsis* counterparts. Like AtAS1, AtLOF1 and AtLOF2 also belong to MYB transcription factor family. The phenotypes of their overexpression lines are similar to *AtAS2*-ovx lines [48], [49]. In this study, it was also examined whether AtLOF1 and AtLOF2 interact with specific AtLBD or MdLBD proteins. The result showed no interaction between AtLOF1/2 and AtAS2 or MdLBD11 (Fig. S4). Therefore, it is presumed that AtLOFs may interact with other LBD proteins or that they have an LBD-independent way to regulate plant development involving lateral organ formation.

Overall, our work indicates that *LBD* genes are conservative in structure and function in different species. By utilizing the apple genome database, it is now feasible to analyze this gene family and predict its function with bioinformatics approaches, which should be helpful for breeding and cultivation to improve agronomic traits such as shoot branching, root growth, nutrient assimilation and uptake in apple.

## Materials and Methods

### Ethics Statement

No specific permits were required for the described field studies. The location is not privately-owned or protected in any way, and the field studies did not involve endangered or protected species.

### The Identification of *MdLBD* Genes in Apple

Two approaches were used to identify the members of the *MdLBD* gene family in apples. First, all known *Arabidopsis LBD* gene sequences were used in a query to perform multiple database searches against the proteome and genome files that were downloaded from the Apple GFDB database (Apple Gene Function and Gene Family Database: http://www.applegene.org/) as well as the GDR database (Genome Database for *Rosaceae*: http://www.rosaceae.org/) [50]. Stand-alone versions of BLAST (Basic Local Alignment Search Tool: http://blast.ncbi.nlm.nih.gov) [51], which are available from the NCBI, were used with an e-value cutoff of 1e-003. All of the protein sequences that were derived from the selected *MdLBD* candidate genes were examined with the domain analysis programs Pfam (Protein family: http://pfam.sanger.ac.uk/) [52] and SMART (Simple Modular Architecture Research Tool: http://smart.embl-heidelberg.de/) [53] with the default cutoff parameters. Second, we analyzed the domains of all of the apple peptide sequences using an HMM (Hidden Markov Model) [54] analysis while searching Pfam. Then, we obtained the sequences by using the PF03195 Pfam number, which contained a typical *MdLBD* domain, from the apple genome sequences by making use of a Perl-based script. Finally, all of the protein sequences were compared with known *MdLBD* sequences by applying ClustalX (http://www.clustal.org/) to verify that the sequences were candidate *MdLBDs* [55].

The isoelectric points and protein molecular weights were obtained with the help of the proteomics and sequence analysis tools on the ExPASy proteomics server (http://expasy.org/) [56]. The chromosomal locations were found in the GDR database by using a Perl-based program.

### The Chromosomal Location and Structure of the *MdLBD* Genes

The chromosomal locations and gene structures were retrieved from the apple genome data that were downloaded from the GDR database. The remaining genes were mapped to the chromosomes with MapDraw [57], and the gene structures of the *MdLBD* genes were generated with the GSDS (http://gsds.cbi.pku.edu.cn/) [58].

### Sequence Alignment and Phylogenetic Analysis

The *MdLBD* sequences were aligned in the ClustalX program with BLOSUM 30 as the protein weight matrix. The MUSCLE program (version 3.52) was also applied to perform multiple sequence alignments in order to confirm the ClustalX result [55], [59]. Phylogenetic trees for the MdLBD protein sequences were constructed with the NJ (neighbor-joining) method of the MEGA5 program (http://www.megasoftware.net/), as well as the p-distance for complete deletion option parameters. The reliability of the trees was tested using a bootstrapping method with 1000 replicates. The images of the phylogenetic trees were drawn in MEGA5 [60].

### Expression Prediction of the *MdLBD* Genes Based on the Expression Profile of *Arabidopsis LBD* Genes

Microarray expression data from various datasets were obtained by making use of Genevestigator (https://www.genevestigator.com/gv/) with the *Arabidopsis* GeneChip platform (Fig. S1) [61]. We performed a local BLASTP program search in BioEdit against the *Arabidopsis* peptide database to find homologous genes by using the putative *MdLBD* genes as queries. Based on the Genevestigator selections, we obtained expression patterns that are presented as heat maps in red/green coding, which reflected the log ratio with red indicating up-regulation and green indicating down-regulation (probe sets in a 22 k Affymetrix GeneChip). A local BLASTN search against existing ESTs (which were downloaded from NCBI, 324742 records; 6 Feb 2012) was conducted to find the corresponding record for each member of these putative *MdLBDs*.

### Plant Materials and Treatments

The apple ‘tea crabapple’ (*Malus hupehensis* Redh. var. pingyiensis) seedlings were used for gene isolation and expression analysis. They were kept at 25°C under long-day conditions (16 h light/8 h dark) in a sand culture. Expression levels for different tissues, leaves, flowers, fruits and roots were collected from a 5-year-old ‘Gala’ apple tree that was grown in natural conditions in the Shandong Province of China. One month-old sand culture seedlings were used for the gene expression analysis. The seedlings were treated with 15 mM KNO_3_ (15 mM KCl was used as a control), hypoxia and 100 µM of different kinds of hormone solutions including ABA, NAA, 6-BA, GA and BR with a 0.05% DMSO control. The treated plant materials were frozen in liquid nitrogen for RNA extraction. *Arabidopsis* plants were planted in the culture room under SD (8 h light/16 h dark) for a month and then moved to LD conditions (16 h light/8 h dark) at 20–22°C.

### RNA Extraction, cDNA Synthesis and Gene Expression Analysis

Total RNA of apple was extracted with the hot borate method as described in our previous report [62], and total RNA of *Arabidopsis* was extracted with Trizol reagent (Invitrogen, USA). Two micrograms of total RNA was used to synthesize first-strand cDNA using the PrimeScript First Strand cDNA Synthesis Kit (Takara, China). RT-PCR was conducted by using cDNA templates to detect the gene expression level. Apple *ACTIN* and *Arabidopsis ACTIN* genes were employed as controls, and the analysis of each type of sample was repeated three times. The specific primers that were used for PCR analysis are listed in Table S1.

### Construction of *MdLBD11* Overexpression Vector

The full length cDNA of *MdLBD11* was identified from the ‘Gala’ apple. The cDNA was ligated into a PMD18-T vector (Takara, China) and then digested with BamHI and cloned into a pBIN vector.

### Yeast Two-hybrid (YTH) Assays

A Gal4-based two-hybrid system was used as directed by the manufacturer (Clontech, USA). The full-length encoding regions of *MdLBD11*, *MdLBD11L* and *AtAS2* were ligated into the DNA binding domain vector pGBKT7 to make the bait plasmid vector. The coding sequence of *AtAS1*, *AtLOF1* and *AtLOF2* were fused to the two-hybrid activation domain vector (pGADT7) as prey. The primers that were used to create these constructs are listed in Table S1. Each pGADT7 vector was separately co-transformed with pGBKT7 vector into yeast strain Y2H for a yeast two-hybrid test. Positive colonies were selected on SD/−Trp-Leu-His-Ade medium. The positive clones of the complexes were selected and assayed for X-gal activity.

### *Arabidopsis* Transformation

Wild type (WT) *Arabidopsis* ecotype Columbia (Col 0) was used. *MdLBD11* was introduced into the WT (Col 0) using the floral dip method and mediated with *Agrobacterium* strain *GV3101* [63]. The seeds of positive transgenic plants carrying the *MdLBD11* constructs were individually harvested. Homozygous transgenic lines were used for further investigation.

## Supporting Information

Figure S1 **Alignment of conserved MdLBD domain sequences.**
(TIF)

Figure S2 **Expression analysis of** ***AtLBD*** **genes based on microarray data from Genevestigator.**
(TIF)

Figure S3 **The phylogenetic analysis and sequence alignment of** ***MdLBD11*** **,** ***MdLBD11L*** **with the** ***Arabidopsis*** **corresponding genes.** A. Phylogenetic analysis of *MdLBD11*, *MdLBD11L* with the *Arabidopsis* corresponding genes. B. Sequence alignment of *MdLBD11*, *MdLBD11L* with the *Arabidopsis* corresponding genes.
(TIF)

Figure S4 **Yeast two-hybrid assay.** Yeast strains containing pGAD-AtAS1, pGAD-AtMYB117 and pGBD-MdLBD11L were assayed for LacZ expression, while pGAD with pGBD-MdLBD11 were used as a negative control.
(TIF)

Figure S5 **Tissue expression from** ***AtLBD*** **genes as acquired from Genevestigator.**
(TIF)

Table S1 Primers for gene clone, vector construction and expression analysis.
(DOC)
