# Supplementary figures and images for: A Genome-Wide Analysis of the LBD (LATERAL ORGAN BOUNDARIES Domain) Gene Family in Malus domestica with a Functional Characterization of MdLBD11
Source: PLoS One. 2013 Feb 28;8(2):e57044. doi: 10.1371/journal.pone.0057044 (PMC3585328; doi:10.1371/journal.pone.0057044)

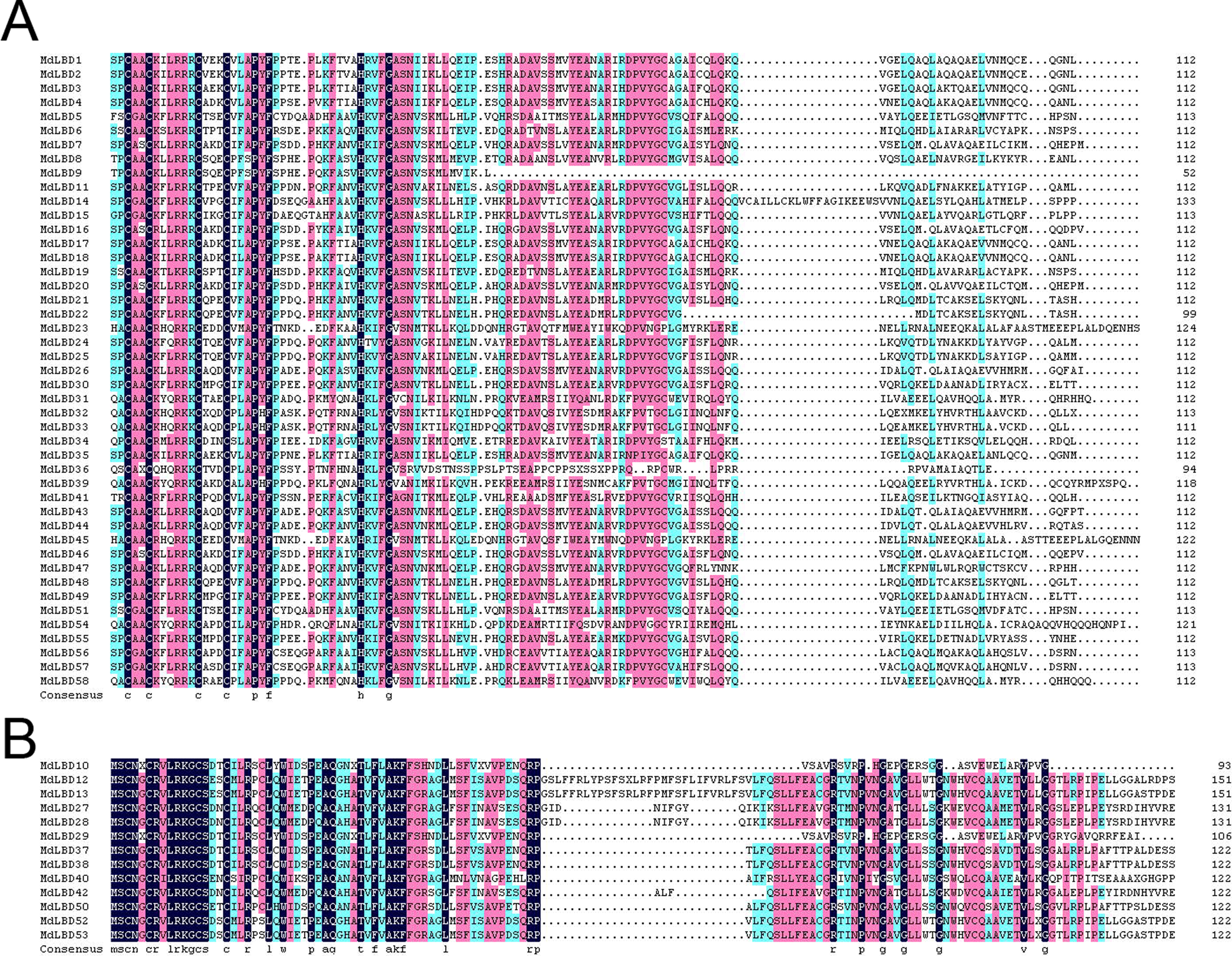

Supplement: Figure S1 — Alignment of conserved MdLBD domain sequences. (TIF) [file pone.0057044.s001.tif]

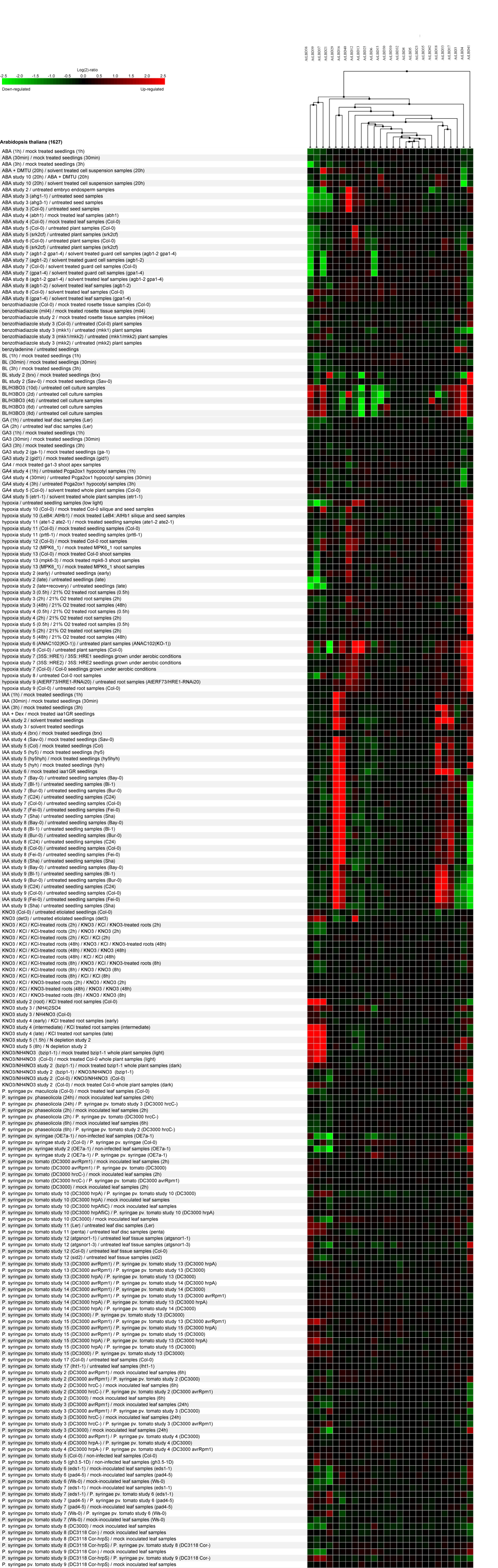

Supplement: Figure S2 — Expression analysis of AtLBD genes based on microarray data from Genevestigator. (TIF) [file pone.0057044.s002.tif]

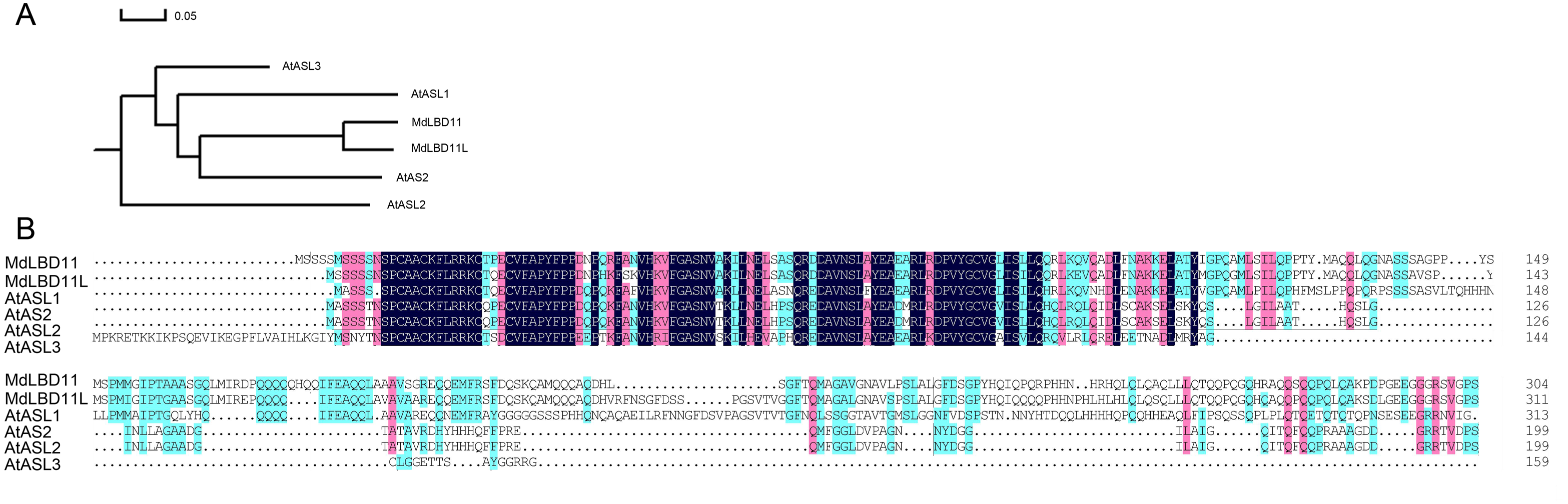

Supplement: Figure S3 — The phylogenetic analysis and sequence alignment of MdLBD11 , MdLBD11L with the Arabidopsis corresponding genes. A. Phylogenetic analysis of MdLBD11, MdLBD11L with the Arabidopsis corresponding genes. B. Sequence alignment of MdLBD11, MdLBD11L with the Arabidopsis corresponding genes. (TIF) [file pone.0057044.s003.tif]

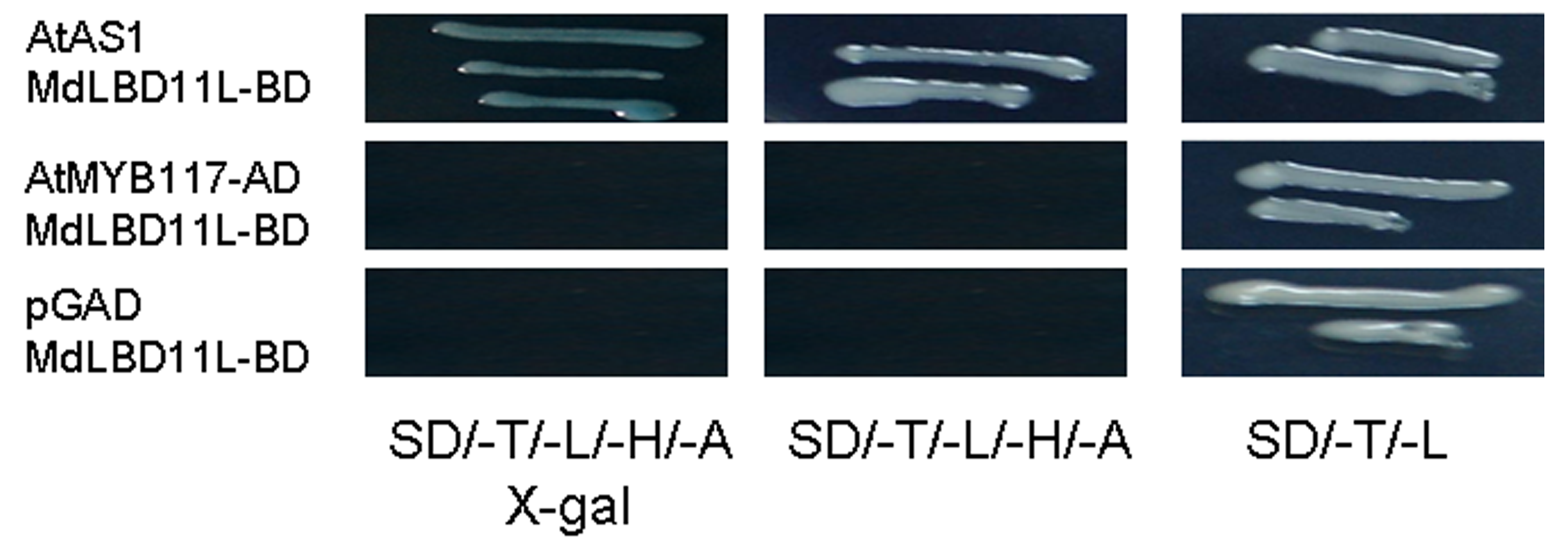

Supplement: Figure S4 — Yeast two-hybrid assay. Yeast strains containing pGAD-AtAS1, pGAD-AtMYB117 and pGBD-MdLBD11L were assayed for LacZ expression, while pGAD with pGBD-MdLBD11 were used as a negative control. (TIF) [file pone.0057044.s004.tif]

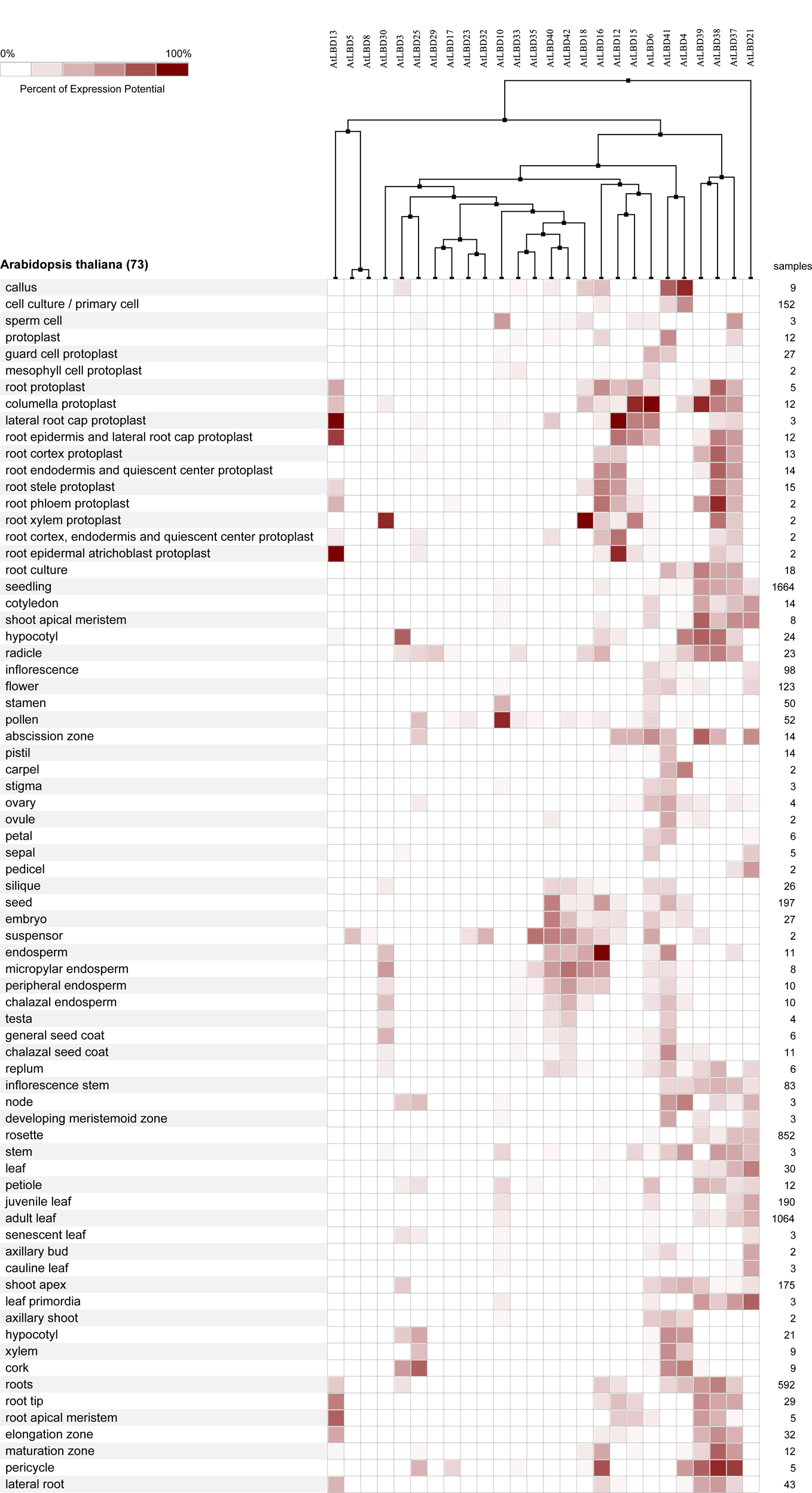

Supplement: Figure S5 — Tissue expression from AtLBD genes as acquired from Genevestigator. (TIF) [file pone.0057044.s005.tif]
