# Supplementary material for: A Genome-Wide Analysis of the LBD (LATERAL ORGAN BOUNDARIES Domain) Gene Family in Malus domestica with a Functional Characterization of MdLBD11
Source: PLoS One. 2013 Feb 28;8(2):e57044. doi: 10.1371/journal.pone.0057044 (PMC3585328; doi:10.1371/journal.pone.0057044)
Supplement: Table S1 — Primers for gene clone, vector construction and expression analysis. (DOC) [file pone.0057044.s006.doc]

**Table S1** Primers for gene clone, vector construction and expression analysis.

| Primer name | Sense primer 5’-3’ | Antisense-primer 5’-3’ |
| --- | --- | --- |
| Primers for gene expression analysis | | |
| STM | CTTCCTCTTGTGCTCCTGC-3 | GGTGCTCCAACCTTCTGAC-3 |
| KNAT6 | ACTCTCAGTAGCCGTCTCC-3 | GCTCCAAAGCAAGAAGAAGG-3 |
| KNAT2 | CGCTTCTCATCCTTTGTATCCTC-3 | ATGGTTCTCTCGCTGAATCTCTTC |
| KNAT1 | ACTAACAACAACAATCATCACCACC | ATGGCTTCAACATCGCTTACG |
| MdARF7 | AGACTGAAGAGGCAGGAGT-3 | TGTATCTGGAAATGGGAGCC |
| MdABI5 | CAATCACACCAACACTTCCAC | ACCAAACTTCATCCACCGTT |
| MdLBD5 | CCTGTGTATGGATGTGTCTCA | AGTGAATGTGTTGCTGCT |
| MdSnRK1 | AATGACCATCCCTGAGATTCGT | GTGTCTGGTGGAGAAACAGC |
| MdLBD6 | GCTGGCTTATGAGGCCGAAGCA | ATGACGACGACGATGAGGG |
| MdLBD8 | TATGAAGCAAATGTGAGGCT | AGGAAGCAACGGAAACAG |
| MdLBD9 | GGAGAGATTTGATGAGGTAGG | GAGAGAAGTAAGGAGAGAAGG |
| MdLBD11 | TTCATATCGTCTGCATACAACTGG | TCTCTCTCCTTCCTCAAAACTCAAA |
| MdLBD13 | GACATCTCCGAACGACAAGAACAA | CAAACAACAAGGACTGAAACAGAAC |
| MdLBD14 | TGGTCGGTGGTGAATCTG | ATCGTATGAAGCGGGCAT |
| MdLBD16 | CTGTGAGCAGTTTGGTTTAT | CATGATCTTGATGTTGATGA |
| MdLBD18 | GGCAACCAGACCAACTTAGGC | GATGTTCCCTCCCTCTTGTTCAAAC |
| MdLBD19 | TTGCCTACGAGGCAGAAGCG | ACAAGAAGAAGAGCACGAC |
| MdLBD26 | GCTGTCCCTTTATGCGTTTCGT | AGCACCTTGTCTTCTACCAGTCTC |
| MdLBD27 | GAGCCACTTCCCGAGTAT | TCGTCGTTTCCGAGTCTT |
| MdLBD37 | CTCTCACTCCCTCCCCTCAA | ATCAGCCTCCCTCCAAAACA |
| MdLBD38 | CTCTCACTCCCTCCCCTCAA | ATCAGCCTCCCTCCAAAACA |
| MdLBD40 | GCCGATTACGCCGATTACTTCC | TTGGGCTTGACAACGGACCG |
| MdLBD42 | GGCGATAGAGACGGTGTT | CTTCTTCTTGGTGGATTGC |
| MdLBD44 | CACAAGCATTCCAAGTTATCCG | GCTCTCCCAAGTCACCAT |
| MdLBD46 | TGTGAGCAGTTTGGTTTATG | TTCTGGTCATGATCTTGATGATG |
| MdLBD47 | CTCAAAGTGCGTCAGACC | ACTCTCCCATTCCCAAGC |
| MdLBD51 | ACTCCAACAACAGGTCGCG | TGTGTGTCAACAGCATCAT |
| MdLBD55 | CCTGTCTACGGATGTGTTG | TCCTCCACGAACCCTACT |
| MdLBD56 | ACTGGCTCACCAGAGCCTT | AGAACTCCTCTCTGCTGC |
| MdACTIN | ACACGGGGAGGTAGTGACAA | CCTCCAATGGATCCTCGTTA |
| AtACTIN | TTTGGAGCCTGGGACTATGGAT | ACGGGGGAATGGGATGAGAT |
| Primers for full length cDNA amplification | | |
| MdLBD11 | TCCGTTGCTGTCGATCCTGCGAA | GGGCAATAGATAATCCAAGGG |
| Primers for construction of yeast-two-hybrid vectors | | |
| MdLBD11(JM) | GGAATTCATGTCGTCGTCCTCAATGT | CGTCGACCAACAAGAGGGACCCACAC |
| MdLBD11L | CGAATTCATGTCGTCGTCGTCGTC | CGTCGACACAAGAGGGACCCACACT |
| AtAS1 | CGTCGACAGTTATACTTGCTCCTGG | CAGATCTCACGTCCACAATGAACATG |
| AtAS2 | CGAATTCATGGCATCTTCTTCAAC | CGTCGACTCAAGACGGATCAACAG |
| AtLOF1 | CGAATTCGAGATGGTGCATGCTGACG | CGTCGACTCACACCGTCCCCAACC |
| AtLOF2 | CGAATTCTCCATGTTTATAACGG | CGTCGACCTATAGGCTACCTTGAAAG |
